# Gender Differences in the Prevalence of Depression among the Working Population of Nepal

**DOI:** 10.1155/2018/8354861

**Published:** 2018-10-28

**Authors:** Ojaswee Sherchand, Nidesh Sapkota, Rajendra Kumar Chaudhari, Seraj A. Khan, Jouslin Kishor Baranwal, Apeksha Niraula, Madhab Lamsal

**Affiliations:** ^1^Department of Biochemistry, B.P. Koirala Institute of Health Sciences, Dharan, Nepal; ^2^Department of Psychiatry, B.P. Koirala Institute of Health Sciences, Dharan, Nepal

## Abstract

**Objective:**

To estimate the prevalence of depression in the working population; to examine if any gender disparity prevails; and to determine the sociodemographic mediators of depression.

**Methods:**

Data from previous research was retrieved for this study. Only paid workers were selected (n=160). Sociodemographic variables including prevalence of moderate depression were compared between the genders using Chi square test. Significant variables were subject to logistic regression. Validated Nepali version of the Beck Depression Inventory scale (BDI-Ia) was used to determine depressive symptoms with a cutoff score of ≥20 considered as moderate depression.

**Result:**

The overall prevalence of moderate depression was 15%, with higher prevalence among working women compared to men [*χ*^2^ (df) = 6.7(1),* P*=0.01], those practicing religions other than Hinduism [*χ*^2^ (df) = 5.5(1),* P*=0.01], those educated up to primary school compared to other education criteria [*χ*^2^ (df) = 9.4(4),* P*=0.03], those having vitamin D deficiency compared to others [*χ*^2^ (df) = 8.5(3),* P*=0.03], and sedentary lifestyle compared to active lifestyle [*χ*^2^ (df) = 6.7(1),* P*=0.009]. The OR (95% CI) for moderate depression was significantly higher in women than in men [3.2 (1.1-9.6),* P= 0.03*] and sedentary lifestyle [2.9(1.1-8.2),* P= 0.04*] even after adjusting for confounding variables.

**Conclusion:**

Working women have increased odds of depression compared to men. Among various characteristics, sedentary lifestyle was the most important causative factor for depression among women.

## 1. Introduction

Over the past decade, there has been a steady increase in the proportion of women joining the labour force and at present Nepal has the third highest women labour force in the world [1]. While this participation of women in paid work brings economic empowerment, it brings forth a new challenge of balancing family and work. The pressure to fulfill each responsibility can act as a mediator of stress and mental disorder, the most common one being depression.

Numerous studies have acknowledged the relationship between depression and work [2–4]. The prevalence of depression in work place has been steadily increasing and was found to vary across occupations. Depression is highly prevalent in Nepal accounting for the second highest rate of “disability adjusted life years” in the world [5]. However, we have insufficient data regarding the prevalence of depression in the Nepalese working population [6, 7]. Employed people often hide depression due to the presumption that the prejudice surrounding depression may cost them their job. The fear of being labeled as “mad” by the society can be taunting and even more so in women who are considered inferior to men in our patriarchal culture [8]. Furthermore, somatization may render the person unaware of the hidden mental disorder making them seek help for these symptoms alone leaving depression untreated [9, 10]. Indeed, depressive symptoms are significantly associated with physical work-related symptoms in indoor workers [11] with musculoskeletal symptoms in health social care workers [12] and with skin symptoms in health care workers [13].

In this study, we estimate the prevalence of depression in the working population in Nepal; examine if there is any disparity in the prevalence of depressive symptoms between the genders; and determine sociodemographic mediators of depressive symptoms between them. We have used the term “working” to denote those individuals who hold paid employment.

## 2. Methods

### 2.1. Sample

**Figure 1:**
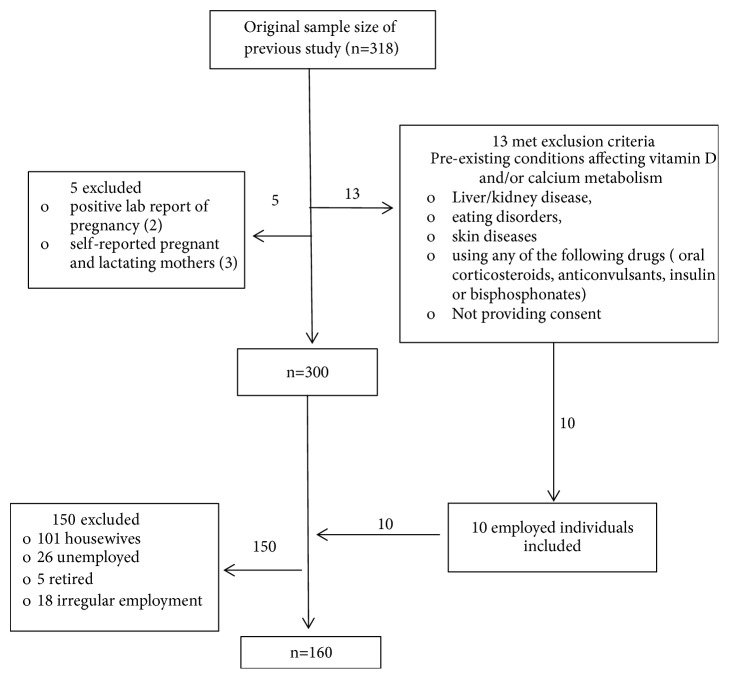
Flowchart of the method applied for sample selection from previous study.

This paper is based on the data obtained from previous study [14] done in B.P. Koirala Institute of Health Sciences, Dharan, Nepal, in the year 2017. Only those individuals holding paid employment were selected while excluding people who were unemployed, retired, and housewives. We also included data from those participants who had met the exclusion criteria in the previous study (hypertensive, diabetic participants) but were engaged in paid employment (Figure 1). Ethical approval was received from Institutional Review Committee of B.P Koirala Institute of Health Sciences.

## 3. Study Variables

### 3.1. Sociodemographic Characteristics

The study population was divided into two main groups: working men and working women. Sociodemographic variables were compared between the two groups (Table 1). The variables included age groups in years (21-40 and 41-65), ethnicity (Brahman and Chhetri, Newar, Janjati, and occupational caste [Dalit]) [15], religion (Hindus and others [Buddhist, Kirat, Muslims, Christians, and Prem Dharma], occupation (legislators, officials, and managers; professionals; technicians, and associate professionals; office assistance, clerk; service workers and shop and market sales workers; skilled and semiskilled agriculture forestry and fishery workers; craft and related trades workers; elementary occupations) [16], education (above higher secondary school, secondary school, primary school, no formal education), marital status (married, unmarried, marital discord [divorced/separated, in conflict with spouse or with in-laws]), family type (alone, nuclear family [single married couple with unmarried children], joint family [married couple living with married children or three different generations living together], socioeconomic status (lower middle and lower class, upper middle and upper class) [17], lifestyle (sedentary: <30 minutes of physical activity [<3 times/week], active: ≥30 minutes of physical activity [≥3 times/week]) [18], and presence of physical comorbidities (none, endocrine disorders, vitamin D deficiency, and pain) (Table 1). The details of vitamin D estimation have been described in previous literature [14].

### 3.2. Anthropometric Measurement

The body weight (kg) and body height (cm) of the participants were measured using standardized technique. Body Mass Index (BMI) was calculated using weight (kg) divided by height (m^2^). In accordance to the World Health Organization recommendations for Asians, BMI was categorized as normal weight (18.5–22.9kg/m^2^), overweight (≥23.0–24.99kg/m^2^), and obese (≥25kg/m^2^) [19].

### 3.3. Assessment of Depressive Symptoms

The Nepali version of Beck Depression Inventory (BDI Ia) scale was used to assess depressive symptoms. This is a validated tool for accurately assessing depressive symptoms with sensitivity of 0.73 and specificity of 0.91 at a cut-off score of 20 signifying moderate depression requiring clinical intervention [20].

### 3.4. Statistical Analysis

Data was entered and analyzed using Statistical Package of Social Science (SPSS) version 11.5. The sociodemographic characteristics of the study sample were expressed using descriptive statistics. Statistical comparison of sociodemographic characteristics among working men and working women was done using *χ*^2^ test. A probability P<0.05 was considered statistically significant. Sociodemographic characteristics were also compared among participants with BDI(Ia) ≥ 20 and < 20 using *χ*^2^ test. Those found to be statistically significant were further subject to logistic regression to calculate adjusted odds ratio (OR) and 95% confidence interval (CI) for BDI(Ia) ≥ 20 [moderate depression] and < 20). *χ*^2^ test was also performed between gender and moderate depression using control variables to find which characteristic of working women and men had higher prevalence of moderate depression. The combined effect of gender and lifestyle on presence of moderate depression was analyzed using relative excess risk due to interaction (RERI) and attributable proportion due to interaction (AP). We calculated RERI and AP using the formulas RERI=OR11−OR10−OR01+1 and AP=(OR11–OR10–OR01 +1)/OR11 [21]. Regression coefficients and the asymptotic covariance matrix from multinomial logistic regression analyses were used to calculate the 95% CIs using delta method described by Anderson [22]. We considered Interaction to be significant if the 95% CI did not include 0.

## 4. Result

The sociodemographic variables between working men and women are presented in Table 1.

**Figure 2:**
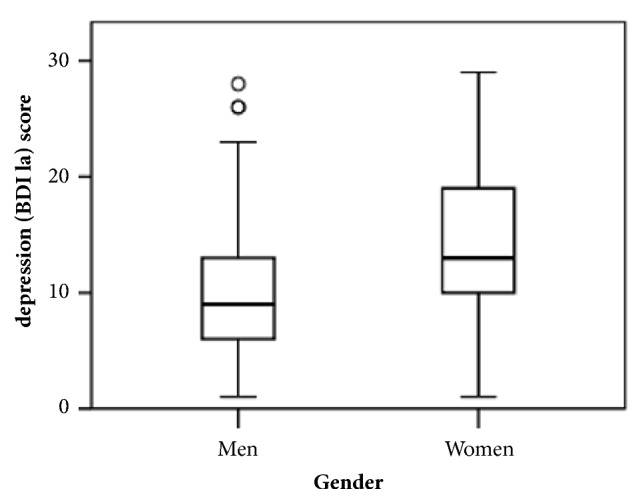
Box-and-whisker plot showing depression scores between men and women. Shown in each boxplot, the median (middle solid line), the 25th and 75th percentile (lower and upper hinge), and 5th and 95th percentiles (whiskers). The width of the box plot is scaled based on number of counts. The median and 25th and 75th percentile of depression (BDI 1a) score in male and female were 9 (6,13) and 13 (10,19), respectively, p< 0.001 (Mann–Whitney test).

Out of 160 participants, 49% were men and 51 % were women. Both groups were comparable without any statistically significant difference in the following categories: age, ethnicity, religion occupation, education, BMI, marital status, socioeconomic status, sedentary lifestyle, and presence of physical comorbidity. However, we found higher prevalence of moderate depression among working women compared to working men (*P = 0.01)* (Table 1). The median depression BDI 1(a) score was significantly higher in women than men (Figure 2).

The overall prevalence of moderate depression was 15%, with higher prevalence among working women compared to men [*χ*^2^ (df) = 6.7(1),* P*=0.01], those practicing religions other than Hinduism [*χ*^2^ (df) = 5.5(1),* P*=0.01], those educated up to primary school compared to other education criteria [*χ*^2^ (df) = 9.4(4),* P*=0.03], those having vitamin D deficiency compared to others [*χ*^2^ (df) = 8.5(3),* P*=0.03], and those with sedentary lifestyle compared to active lifestyle [*χ*^2^ (df) = 6.7(1),* P*=0.009] (Table 2).

The frequency of moderate depression among gender characteristics found higher prevalence of moderate depression among women of age group 41-65 years compared to men of the same age group [*χ*^2^ (df) = 7.6(1),* P* =0.009], women practicing religions other than Hinduism compared to men [*χ*^2^ (df) = 9.9(1),* P*=0.002], Janajati women compared to Janajati men [*χ*^2^ (df) = 7.4(1),* P*=0.007], women living in nuclear family compared to men living in nuclear family [*χ*^2^ (df) = 4.8(1), p=0.03], married women compared to married men [*χ*^2^ (df) = 5.6(1),* P*=0.01], women with sedentary lifestyle compared to men with sedentary lifestyle [*χ*^2^ (df) = 7.4(1), p=0.006], women with endocrine disorders compared to men with the same [*χ*^2^ (df) = 5.9(1),* P* =0.03], women with lower middle and lower socioeconomic class compared to men with the same socioeconomic status [*χ*^2^ (df) = 4.2(1), p=0.04] (Table 3).

Logistic regression analysis tested for characteristics found to be significant on Chi square test (Table 2) revealed the OR (95% CI) for moderate depression was significantly higher in women compared to men [3.2(1.1-9.6),* P= 0.03*] and those having sedentary lifestyle [2.9(1.1-8.2),* P= 0.04*] even after adjusting for religion, education, and physical comorbidities (Table 4). The interaction between gender, lifestyle, and moderate depression revealed significantly higher odds of moderate depression among sedentary women, OR (95% CI) [8.3(1.9-36),* P=* 0.005]. The joint effect of gender and life style on moderate depression showed positive additive interaction; however they were not significant [RERI=5.2(-3.8-14), the AP due to this interaction = 0.6(0.1-1.1)] (Table 5).

## 5. Discussion

Depression is a major challenge to the economic development of the country as it primarily affects the working population [23]. Depression leads to decline in cognitive abilities and interpersonal skills, lack of motivation, and disregard to safety measures [24] which translates to decreased work performance, and absenteeism directly impacting the labour force market. Moreover, untreated depression can lead to unemployment, poverty, substance abuse, and suicide which add further to the economic burden of the disorder [25].

In our study, we found significantly higher prevalence of moderate depression among working women compared to working men. This was in agreement to a study conducted in Italy where female radiologists had a higher rate of depression disorder compared to male colleagues [26]. Furthermore, when marital status was examined in our study, married women had significantly higher prevalence of depression compared to married men. The same did not hold true for unmarried and marital discord categories. In the context of Nepalese society, men are often exempt from domestic responsibilities while women are expected to look after the family and perform household chores even if they are employed [27, 28]. The additional burden of unpaid work means women have to work longer hours than men which can cause mental and physical exhaustion.

Socioeconomic status (SES) did not affect the prevalence of depression. This finding was different from previous studies reporting lower socioeconomic status (LSES) as a risk factor for depression [29–31]. However, on examining each socioeconomic class among gender category, we observed women from lower middle and lower classes had significantly greater prevalence of moderate depression compared to men of the same socioeconomic status. This was similar to a study conducted in Belgium reporting higher effects of SES on depressive symptoms among women [31]. The reason behind such disproportional effect of socioeconomic status on gender remains unresolved in our study. We can only postulate it may be due to the way men and women handle everyday stressors as economic hardships associated with LSES.

The prevalence of moderate depression was higher among individuals with sedentary lifestyle compared to active people. When examining genders we found that sedentary women had significantly higher rates of depression irrespective of which SES they belonged to. Another important finding was the effect of religion on the prevalence of depression. Those practicing Hinduism was buffered against depression. The mechanism behind such occurrence needs further insight into the religious and cultural aspects between Hinduism and other religions and how they affect the minds of the people.

People suffering from vitamin D deficiency had greater prevalence of moderate depression than those with other comorbidities. However, on comparing between the genders, the effect of vitamin D did not vary; instead we found women suffering from endocrine disorders had higher occurrence of depression compared to men with the same.

Logistic regression showed significant association of gender and lifestyle with moderate depression even after adjustment for confounding variables. The odds of depression were 3.2 times higher in women than in men and 2.9 times higher in people with sedentary lifestyle compared to those with active lifestyle. Among various characteristics of women, sedentary lifestyle was significantly associated with depression. The combined effect of gender and lifestyle on presence of moderate depression was additive but not significant. Our study highlights the need to identify factors including workplace dynamics contributing to gender disparity in the prevalence of depression. Furthermore, it urges the need to initiate workplace screening and intervention programs to prevent and treat depression.

## 6. Conclusion 

We found the gender of women and sedentary lifestyle are independently associated with higher risk of moderate depression. Working women have threefold greater risk of developing depression than working men.

## Figures and Tables

**Table 1:** Comparison of sociodemographic variables between working men and working women.

		Total n(%)	Men	Women	*P*
n(%)^a^		160(100)	79(49)	81(51)	

**Age groups** ** (years)**	21-40	95(59)	44(27)	51(32)	0.3
	41-65	65(41)	35(22)	30(19)	

**Religion**	Hindu	118(74)	58(36)	60(38)	0.9
	Others	42(26)	21(13)	21(13)	

**Ethnic groups**	Brahman and Chhetri	62(39)	37(23)	25(16)	0.2
	Newar	27(17)	12(8)	15(9)	
	Janajati	56(35)	23(14)	33(21)	
	Occupational caste (dalit)	15(9)	7(4)	8(5)	

**Occupation**	Legislators, Officials & Managers	15(9)	10(6)	5(3)	0.1^b^
	Professionals	29(18)	12(7)	17(11)	
	Technicians and associate Professionals	11(7)	8(5)	3(2)	
	Office assistance, clerk	4(3)	4(3)	0(0)	
	Service workers & shop & market sales workers	36(23)	15(10)	21(13)	
	Skilled and semi-skilled agriculture forestry and fishery workers	44(27)	20(12)	24(15)	
	Craft and related trades workers	12(7)	7(4)	5(3)	
	Elementary Occupations	9(6)	3(2)	6(4)	

**Education**	Above Higher Secondary School	49(31)	26(16)	23(15)	0.2
	Higher Secondary School	15(9)	10(6)	5(3)	
	Secondary School	47(29)	25(15)	22(14)	
	Primary School	27(17)	10(6)	17(11)	
	No formal education	22(14)	8(5)	14(9)	

**BMI**	Normal	56(35)	31(19)	25(16)	0.2
	Overweight	78(49)	39(24.5)	39(24.5)	
	Obese	26(16)	9(6)	17(10)	

**Family Type**	Alone	24(15)	7(4)	17(11)	0.01
	Nuclear family	77(48)	35(22)	42(26)	
	Joint family	59(37)	37(23)	22(14)	

**Marital Status**	Married	128(80)	66(41)	62(39)	0.3
	Unmarried	10(6)	3(2)	7(4)	
	Marital discord	22(14)	10(6)	12(8)	

**Socioeconomic ** **Status**	Lower middle and lower class	107(67)	51(32)	56(35)	0.5
	Upper middle and upper class	53(33)	28(17)	25(16)	

**Lifestyle**	Active	92(58)	46(29)	46(29)	0.8
	Sedentary	68(42)	33(20)	35(22)	

**Depression scores**	BDI (Ia) score < 20	136(85)	73(46)	63(39)	0.01
	BDI (Ia) score ≥ 20 (moderate depression)	24(15)	6(4)	18(11)	

**Physical ** **Comorbidities**	None	81(50)	42(26)	39(24)	0.9
	Endocrine disorders	21(13)	10(6)	11(7)	
	Vitamin D deficiency	33(21)	16(10)	17(11)	
	Pain	25(16)	11(7)	14(9)	

All values are expressed as number (percentage) [n(%)] of rows exception n(%)^a^ where values are expressed as number (percentage) [n(%)] of columns. Comparisons between men and women were performed using *χ*^2^ test except ^b^ where Fischer's exact test was used.

**Table 2:** Comparison of sociodemographic variables between groups with (BDI(Ia) ≥ 20) and without (BDI(Ia) <20) moderate depression.

		Total n(%)	BDI(Ia) <20	BDI(Ia) ≥ 20	*P*
n(%)^a^			136(85)	24(15)	

**Age groups (years)**	21-40	95(59)	80(50)	15(9)	0.7
	41-65	65(41)	56(35)	9(6)	

**Working Population**	Men	79(49)	73(45)	6(4)	0.01
	Women	81(51)	63(39)	18(11)	

**Religion**	Hindu	118(74)	105(66)	13(8)	0.01
	Others	42(26)	31(19)	11(7)	

**Ethnic groups**	Brahman and Chhetri	62(39)	53(33)	9(6)	1.00^b^
	Newar	27(17)	23(14)	4(3)	
	Janajati	56(35)	47(29)	9(6)	
	Occupational caste (dalit)	15(9)	13(8)	2(1)	

**Occupation**	Legislators, Officials & Managers	15(9)	12(7)	3(2)	0.08^b^
	Professionals	29(18)	27(17)	2(1)	
	Technicians and associate Professionals	11(7)	11(7)	0	
	Office assistance, clerk	4(3)	4(3)	0	
	Service workers & shop & market sales workers	36(23)	32(20)	4(3)	
	Skilled and semi-skilled agriculture forestry and fishery workers	44(27)	31(19)	13(8)	
	Craft and related trades workers	12(7)	10(6)	2(1)	
	Elementary Occupations	9(6)	0	9(6)	

**Education**	Above Higher Secondary School	49(31)	47(30)	2(1)	0.03^b^
	Higher Secondary School	15(9)	13(8)	2(1)	
	Secondary School	47(29)	39(24)	8(5)	
	Primary School	27(17)	19(12)	8(5)	
	No formal education	22(14)	18(11)	4(3)	

**BMI**	Normal	56(35)	47(29)	9(6)	0.7
	Overweight	78(49)	68(43)	10(6)	
	Obese	26(16)	21(13)	5(3)	

**Family Type**	Alone	24(15)	20(12)	4(3)	0.5
	Nuclear family	77(48)	68(42)	9(6)	
	Joint family	59(37)	48(30)	11(7)	

**Marital Status**	Married	128(80)	109(68)	19(12)	0.2^b^
	Unmarried	10(6)	10(6)	0	
	Marital discord	22(14)	17(11)	5(3)	

**Socioeconomic Status**	Lower middle and lower class	107(67)	88(55)	19(12)	0.1
	Upper middle and upper class	53(33)	48(30)	5(3)	

**Lifestyle**	Active	92(58)	84(53)	8(5)	0.009
	Sedentary	68(42)	52(32)	16(10)	

**Physical Comorbidities**	None	81(50)	72(45)	9(5)	0.03^b^
	Endocrine disorders	21(13)	16(10)	5(3)	
	Vitamin D deficiency	33(21)	24(15)	9(6)	
	Pain	25(16)	24(15)	1(1)	

All values are expressed as number (percentage) [n(%)] of rows except n(%)^a^ where values are expressed as number (percentage) [n(%)] of columns. P value was obtained from *χ*^2^ test except ^b^ from Fischer's exact test.

**Table 3:** Prevalence of moderate depression amongst various characteristics of men and women.

		Moderate depression ^a^			
Variables	Gender	Absent	Present	Total	P value
**Age groups**					
21-40 years	Men	39(41)	5(5)	44(46)	0.2 ^a^
	Women	41(43)	10(11)	51(54)	
41-65 years	Men	34(52)	1(2)	35(54)	0.009 ^b^
	Women	22(34)	8(12)	30(46)	

**Religion**					
Hindu	Men	53(45)	5(4)	58(49)	0.4 ^a^
	Women	52(44)	8(7)	60(51)	
Others	Men	20(48)	1(2)	21(50)	0.002
	Women	11(26)	10(24)	21(50)	

**Ethnic groups**					
Brahman and Chhetri	Men	34(55)	3(5)	37(60)	0.1 ^b^
	Women	19(30)	6(10)	25(40)	
Newar	Men	11(40)	1(4)	12(44)	0.6 ^b^
	Women	12(44)	3(11)	15(55)	
Janajati	Men	23(41)	0	23(41)	0.007 ^b^
	Women	24(43)	9(16)	33(59)	
Occupational caste (dalit)	Men	5(33)	2(13)	7(46)	0.2 ^b^
	Women	8(53)	0	8(53)	

**Family type**					
Alone	Men	7(29)	0	7(29)	0.2^b^
	Women	13(54)	4(17)	17(71)	
Nuclear	Men	34(44)	1(1)	35(45)	0.03^b^
	Women	34(44)	8(10)	42(54)	
Joint	Men	32(54)	5(9)	37(63)	0.3^b^
	Women	16(27)	6(10)	22(37)	

**Marital status**					
Married	Men	61(48)	5(4)	66(52)	0.01 ^a^
	Women	48(38)	14(11)	62(49)	
Marital discord	Men	9(41)	1(5)	10(46)	0.3^b^
	Women	8(36)	4(18)	12(54)	

**Socioeconomic Status**					
Lower middle and lower class	Men	46(43)	5(5)	51(48)	0.04 ^a^
	Women	42(39)	14(13)	56(52)	
Upper middle and upper class	Men	27(51)	1(2)	28(53)	0.1 ^b^
	Women	21(40)	4(7)	25(47)	

**Lifestyle**					
Active	Men	43(47)	3(3)	46(50)	0.7^b^
	Women	41(45)	5(5)	46(50)	
Sedentary	Men	30(44)	3(4)	33(48)	0.006 ^a^
	Women	22(32)	13(19)	35(51)	

**Physical comorbidities**					
None	Men	39(48)	3(4)	42(52)	0.3 ^b^
	Women	33(41)	6(7)	39(48)	
Endocrine disorders	Men	10(48)	0	10(48)	0.03 ^b^
	Women	6(28)	5(24)	11(52)	
Vitamin D deficiency	Men	13(39)	3(9)	16(48)	0.4 ^b^
	Women	11(33)	6(18)	17(51)	
Pain	Men	11(44)	0	11(44)	1 ^b^
	Women	13(52)	1(4)	14(56)	

All values are expressed as number (percentage) [n(%)] of rows.

^a^Moderate depression: cut-off score of BDI I(a) score ≥ 20.

Comparisons of different variables of men and women with prevalence of moderate depression were performed using ^a^*χ*^2^ test or ^b^ Fischer's exact test.

**Table 4:** Multiple logistic regression showing OR (95%) of moderate depression for gender.

		Unadjusted OR (95%CI)	*P value*	Adjusted OR (95%CI)^a^	*P value*
Gender	Men	Reference	0.01	Reference	0.032
	Women	3.4(1.3-9.2)		3.2(1.1-9.6)	

Lifestyle	Active	Reference	0.01	Reference	0.04
	Sedentary	3.2(1.2-8)		2.9(1.1-8.2)	

^a^Adjusted for religion, education, and physical comorbidities.

**Table 5:** Additive interaction derived from OR (95%) of combined effect of gender and lifestyle for moderate depression.

Variables		Unadjusted OR (95%CI)	Adjusted OR (95%CI)^a^
Men(0)			
Active(0)		Reference	
Sedentary(1)		1.4(0.2-7)	1.8(0.3-10)

Women(1)			
Active (0)		1.7(0.3-7)	2.2(0.4-10)
Sedentary(1)		8.4(2-32)	8.3(1.9-36)

RERI (95%CI) ^b^	5.3(-3.8-14)		
AP(95%CI) ^c^	0.6(0.1-1.1)		

^a^Adjusted for religion, education, and physical comorbidities.

^b^Relative Excess Risk due to Interaction (RERI) = (OR11 - OR10 - OR01 + 1).

^c^Attributable proportions due to the interaction (AP) = (OR11 - OR10 - OR01 + 1)/OR11) were derived from the above shown odds ratios (ORs) from logistic regression adjusted for religion, education, and physical comorbidities.

## Data Availability

The data used to support the findings of this study are available from the corresponding author upon request.
